# S247. GENDER DIFFERENCES IN FUNCTIONALITY IN INPATIENT POPULATION AFFECTED WITH SCHIZOPHRENIA AND OTHER PSYCHOSIS

**DOI:** 10.1093/schbul/sby018.1034

**Published:** 2018-04-01

**Authors:** Siddarta Acebillo, Rebeca García Collell, Lourdes Nieto, Carmina Massons, Noèlia Ortuño, Cristina Domènech, Jesus Cobo

**Affiliations:** 1 Corporació Sanitària Parc Taulí; 2 Corporació Sanitària i Universitària Parc Taulí. CIBERSAM, Universitat Autònoma de Barcelona; 3 Instituto Nacional de Psiquiatría Ramón de la Fuente Muñiz; 4 Centre Numància, Parc Sanitari Sant Joan de Dèu

## Abstract

**Background:**

Studies that have examined gender differences in social functioning have found better performance in women but other studies failed to detect these differences (Ochoa et al, 2012).

We aim to study gender differences in functionality in a severe sample of schizophrenia and schizophrenia spectrum disorder patients, and to analyse the relationships between functionality, psychopathological dimensions and gender.

**Methods:**

Multicenter cross-sectional naturalistic study sample of 124 (66.9% men) schizophrenia and non-affective schizophrenia spectrum disorder inpatients from a University Hospital Acute Unit setting. Diagnosis was made following DSM IV-TR. Severity of psychopathology was assessed using Positive and Negative Syndrome Scale (PANSS) Lindenmayer’s Factors (Kay et al., 1987). The deficit of insight and its three dimensions were evaluated by the Scale of Unawareness of Mental Disorders (SUMD) (Amador et al., 1993). Functionality was measured by the Global Assessment of Functioning Scale (GAF) and the Personal and Social Performance scale (PSP) (Morosini et al, 2000). Premorbid Intelligence Quotient (IQ) was estimated by verbal sub-scale of WAIS. Bivariate analysis and parametric correlations were performed in order to make a multiple linear regression model of insight dimensions.

**Results:**

The sample included a 42.7% of people affected of schizophrenia, with a severe psychopatology (mean total PANSS scores 83.7, sd. 23) and different clinical situations. In our sample, there were no significant differences in functionality neither with the GAF or the PSP global scores. Women performed significatively worst in the PSP self-care subscale (p=0.024), and men performed significatively worst in the PSP disturbing and aggressive behaviours subscale (p=0.033).

In the regression analysis, the total sample (men and women) showed a model for the PSP global scores including only the PANSS Lindenmayer’s Desorganized/Cognitive Factor (R2 0.412). PSP self-care subscale showed a model including only PANSS Lindenmayer’s Desorganized Factor (R2 0.319). PSP socially useful activities subscale showed a model including only PANSS Lindenmayer’s Negative Factor (R2 0.213). PSP personal and social relationships subscale showed a model including the PANSS Lindenmayer’s Negative and the Excitatory Factors (R2 0.533). PSP disturbing and aggressive behaviours subscale showed a model including only PANSS Lindenmayer’s Excitative Factor (R2 0.363). Gender and other clinical, sociodemographical or outcome factors did not have influence in the models.

In men sample a model for the PSP global scores included only the PANSS Lindenmayer’s Desorganized Factor (R2 0.511). PSP self-care subscale showed a model including both PANSS Lindenmayer’s Desorganized Factor and IQ (R2 0.585). PSP socially useful activities subscale showed a model including only PANSS Lindenmayer’s Negative Factor (R2 0.394). PSP personal and social relationships subscale showed a model including only the PANSS Lindenmayer’s Negative Factor (R2 0.626). PSP disturbing and aggressive behaviours subscale showed a model including only PANSS Lindenmayer’s Excitative Factor (R2 0.478).

In women in our sample, there were no explicative model. Moreover, global GAF scores were not explicated by a model in our sample.

**Discussion:**

According to our data, men and women seem to be similar in levels of global functionality. Nevertheless, they showed several differences in specific domains of functionality measured by PSP. Model explainig the association of functionality with psychopatological and clinical variables showed a significative relatioship of isolated psychopatological factors and functional domains.

